# Trick or treat? – when children with childhood food allergies lead parents into unhealthy food choices

**DOI:** 10.1186/s12889-020-09556-x

**Published:** 2020-09-25

**Authors:** Ali B. Mahmoud, Dieu Hack-Polay, Leonora Fuxman, Dina Naquiallah, Nicholas Grigoriou

**Affiliations:** 1 University of Wales Trinity Saint David, London, UK; 2 Crandall University, Moncton, Canada; 3 University of Lincoln, Lincoln, UK; 4 St. John’s University, New York, USA; 5 University of Illinois at Chicago, Chicago, IL USA; 6 Monash University, Melbourne, Victoria Australia

**Keywords:** Childhood obesity, Food allergy, Parenting, Attitudes, Gender, Healthy eating, COVID-19

## Abstract

**Background:**

This study examines the relationships between childhood food allergy and parental unhealthy food choices for their children across attitudes towards childhood obesity as mediators and parental gender, income and education as potential moderators.

**Methods:**

We surveyed parents with at least one child between the ages of 6 and 12 living in Canada and the United States. We received 483 valid responses that were analysed using structural equation modelling approach with bootstrapping to test the hypothetical path model and its invariance across the moderators.

**Results:**

The analysis revealed that pressure to eat fully mediated the effects of childhood food allergy and restriction on parental unhealthy food choices for their children. Finally, we found that parental gender moderated the relationship between childhood food allergy and the pressure to eat.

**Conclusions:**

The paper contributes to the literature on food allergies among children and the marginalisation of families with allergies. Our explorative model is a first of its kind and offers a fresh perspective on complex relationships between variables under consideration. Although our data collection took place prior to Covid-19 outbreak, this paper bears yet particular significance as it casts light on how families with allergies should be part of the priority groups to have access to food supply during crisis periods.

## Background

Various factors determine the choice of food. Although a more substantial interest is shown in understanding and determining environmental, social and cultural factors that influence the choice of food, an assessment of the influence of parents in early years and the association with the nutritional health of children are vital. Family eating behaviours, social status and the structure of family alongside cultural heritage are among the factors that influence food choices in a family setting [1]. During the last decade, socio-economic changes profoundly influenced family diets and consequently, food choices for children, which are often high in fat and sugar. The decreased time spent on preparing healthy meals [2, 3] and the increased availability as well as affordability of highly processed foods has contributed to the popularity of convenient but unhealthy alternative foods. That has led to the growing imbalance in the children’s diets, which now contain more calories, fat and sodium than the right proportion advised for their age group [4], thus causing concerns about the growing statistics on childhood obesity. Latest data from the ‘Canadian Community Health Survey’ (StatCan) shows steady yearly increases in the percentage of adolescents who are deemed overweight or obese from 2005 to 2018 based on the Body Mass Index (BMI) [5], reaching 28% in 2018 [5], although showing a somewhat decreased percentage of 23.7% in 2018. Further, in the United States, recent numbers show that childhood obesity has affected 14 million children and adolescents with an almost 19% prevalence [6]. The global overweight and obesity prevalence for children is predicted to reach 9% [7], i.e., 60 million children in 2020.

As a primary cause of increased obesity rates, food decisions are also seen to be linked to other health issues affecting both individuals and society as a whole [8]. Additionally, child food allergy alongside parental attitudes towards childhood obesity has been considered critical factors when parental food choices for children are debated (e.g., [9]). There has been no shortage of research on the causes of childhood obesity and children’s eating habits [10], with oftentimes contradictory outcomes. It has been found, for example, that restricting children’s access to snacks makes restricted foods more attractive [11], hence promoting unhealthy food choices. In contrast, exercising no control over food selections has been shown to contribute to the development of weight excess [12]. Child’s predisposition to food allergies further complicates our understanding of parental food choices for their children.

This study attempts to clarify further complex relationships in parental food selection decisions for their children who experience food allergies, weighting in parental behavioural strategies towards food choices for their kids (such as pressure to eat and restriction). The study is particularly relevant in the current period of the coronavirus (covid-19) outbreak which has led to empty shelves in shops in the United States and Canada for food suitable for allergy sufferers. With an increase in panic buying, families that have children with allergy issues struggle to source essential supplies [13]. This issue suggests that parents of children with allergies are marginalised [14, 15]. The lockdown of whole countries such as the USA and Canada meant that millions of people have had to be in isolation in their homes, which causes a significant number of people to put on weight [2, 16–18]. We develop and test a novel path model that links child food allergy to parental unhealthy food choices for their children via parental attitudes towards childhood obesity. We also examine the moderating effects of a parent’s gender, education, and income [19] on the path model by utilising a structural equation modelling approach and bootstrapping. Our explorative model is a first of its kind to shed more light into complex relationships between variables under consideration.

## Theoretical foundations and hypotheses

Food allergy refers to abnormal reactions to a food protein and is the most common source of critical allergic reactions that pose life-threatening complications [20]. Anaphylaxis is a common form of food allergy. Food allergy is deemed a widespread chronic condition with an expected prevalence of 4–9% in children [20]. About 8% of North American children suffer from a reported food allergy [14, 21, 22]. It is projected that food allergies cost the US economy about USD 25 billion per year, ranging from loss of family workdays, to direct and indirect costs of medical appointments [14, 21, 23]. Food allergies are unique because till recently; there were no pharmaceutical interventions to prevent allergic reactions [24]. As a result, the management of children’s dietary intake through strict avoidance of certain food types remains critical [25]. However, there is now an FDA-approved paediatric peanut allergen immunotherapy “Palforzia” and many food allergen immunotherapies (i.e. oral, epicutaneous, sublingual) that are currently being tested and have been found to be safe/efficacious in phase I-II studies [26]. If these results are repeated in phase III trials, they will constitute a significant breakthrough in food allergy.

Childhood allergy to food is increasingly widespread. It is often accompanied by a heightened level of parental worry about caring for such children [27] due to the need to set and follow dietary restrictions which in turn can significantly influence family living standards, including their social activities [28]. The food-allergy-related fears, coupled with a continued need for food choice monitoring and possible personal activity constraints, can have psychological implications for the people concerned and their caregivers. Notably, it has been shown that parents of food-allergic children are more anxious than parents of children with other health problems [29]. The occurrence of allergy amongst children limits the consumption of nutritional food often without adequate nutrient replacement [30]. In addition to dietary challenges, children with food intolerances also face other issues such as anxiety, food aversion and refusal, which increases the risk of poor nutrition, affecting parental guidance and parental behaviour towards food decisions [31]. Furthermore, children’s food allergies have been found to have a significant effect on parent’s in-home meal preparation [32]. Additionally, research suggests a positive association between increased body weight and predisposition to food allergies [33], while also links parental perceptions of food allergies to their children’ diet [34] which collectively, provide additional insights into the role food allergies play in the food selection process.

In addition to the genetic aspects connected with obesity, a number of behavioural obesity risk factors, such as an unhealthy diet with excessive sodium and fat consumption coupled with the absence of physical exercise have also been highlighted in previous research [35]. Such behavioural obesity risk factors are directly influenced by social and environmental components at home, school, and other social circles [36]. In the context of the ongoing pandemic with covid-19, more evidence is emerging to support the link between children not attending school due to the lockdown of many countries and obesity levels in children [16, 37]. However, the existence of the linkage between parental food decisions and the way they impact behavioural obesity risk factors in children have not been studied before, and that is where this research study offers a novel contribution.

The parental pressure put on children to eat reflects the attitudes of the parent towards obesity. Eating pressuring strategies could be seen as either friendly communicative or correctional when parents use them. An example of correctional is when parents engage in bribing children to eat all the food on their plates [38]. Alternatively, as a friendly communicative strategy, parents may attempt to encourage their children to develop good eating habits and share with them directive information that enhance the chances of making healthy food choices [39]. Most childhood obesity prevention programs are school-based, meaning that the primary understanding of nutrition and healthy eating is acquired at school, not at home [40]. As a result, parents who do not know the dietary requirements and nutritional needs of their children, often put pressure on their children to ‘clean up their food,’ an idea that a meal is not complete unless all food is eaten [40]. Research indicates that eventually, the practice of pressuring kids to eat leads to the development of dislikes for foods that children are told to eat [41]. Such parental eating pressure behaviour primarily links to the cultural beliefs that the absence of hunger and heavier children are associated with a healthier status [42, 43]. Parental pressure eating behaviour has also been linked to obesity development in an obesogenic environment when food scarcity is present [40]. In this study, the pressure to eat is theorised as a negative concept, that is, pressure to eat presents a risk associated with obesity.

Finally, parental restriction on their children’s food intake is another vital variable in our study. According to LC Moore, CV Harris and AS Bradlyn [44], when children are permitted to select their own foods from a variety of both healthy and unhealthy food choices, children typically select unhealthy foods (e.g., high in fat). The Parental restriction is also linked to allergen avoidance for children suffering from a food allergy, but often not replacing eliminated nutrients with proper alternatives [30].

Even worse is the fact that a child is allowed to consume what they like so that children select foods with high levels of sodium, fat and sugar [45]. Some parents believe that they grant their children’s wishes for these foods out of love and see them as treats [46]. Some parents are directed to unhealthy food out of sheer ignorance about diet generally [47, 48]. As a result of these constraints, it is believed that 90% of American families have their children on foods that challenge positive dietary practices that are conducive to good health [46, 47]. Additionally, JS Savage, JO Fisher and LL Birch believe that the relatively inexpensive cost of energy-dense foods is a crucial factor that lures parents towards such choices [48]. In the current covid-19 pandemic, with the spiralling cost of food generally, millions of poor households in the USA and around the world have turned to ‘junk’ food for survival. In the period of the pandemic, more than in any other period, economics, thus, dictates what parents treat their children with despite the consequences of unhealthy food.

**Fig. 1:**
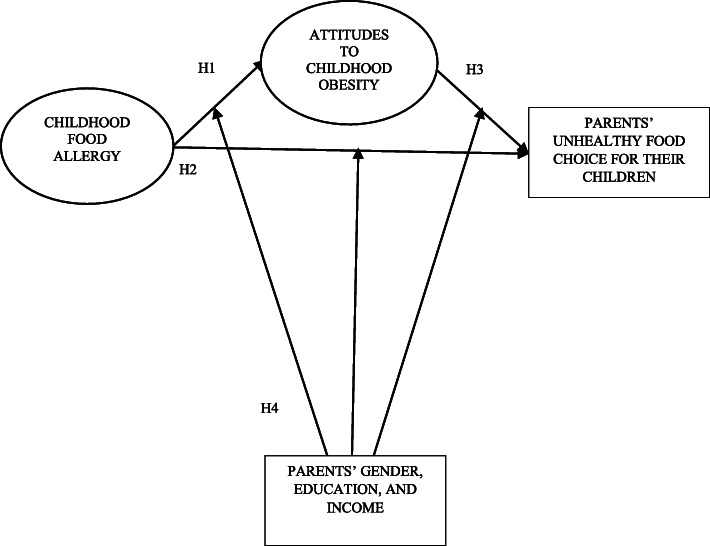
Hypothetical model

Recent research has conceptualised childhood food allergy as a moderating variable for a number of unhealthy parental eating behaviours for their children with suggestions that future research conceptualises childhood food allergy as an endogenous variable when investigating unhealthy eating behaviours [8]. Hence, we hypothesise the following relationships between child food allergy, parental unhealthy food choices and obesity attitudes (see Fig. 1):

- Hypothesis 1: Child food allergy predicts parental attitudes towards childhood obesity.
- Hypothesis 2: Child food allergy predicts parental unhealthy food choices for their children.
- Hypothesis 3: Parental attitudes towards childhood obesity predict unhealthy food choices for their children.

*Hypothesis 3a: Pressure to eat increases the chances for parental unhealthy food choices for their children.*

*Hypothesis 3b: Restriction on children’s dietary intake lowers the chances for parental unhealthy food choices for their children.*

- Hypothesis 4: Parents’ gender, education, and income moderate the relationships stated in Hypotheses 1, 2, and 3.

Also, based on the directions of the relationships in Hypotheses 1, 2 and 3 and according to RM Baron and DA Kenny’s [49] conceptualisation of the properties of mediators, we will test parental attitudes towards childhood obesity as a potential mediator between childhood food allergy and parental unhealthy food choices.

## Methods

The study took place between September 2017 and August 2019. We randomly sampled American and Canadian parents of children aged 6–12, approaching them via their schools and social networks. Eight primary schools were chosen randomly (four schools in Chicago area, US and four schools in a province of Ontario, Canada) where a total of 1000 questionnaires were distributed. To increase the representativeness of our sample, we used a stratified sampling technique. In that regard, fifty questionnaires were allocated to each of the two groups of parents, that is, of students both prone and not prone to food allergies in each school. Proneness to food allergy was determined during an initial phone call with the parents to obtain basic information about the parental history of atopy. The initial phone call also allowed the researchers to gain their prior consent to take part in the study. Two hundred questionnaires were distributed via groups dedicated to child diets on social networks. The survey would not be submitted unless it was fully completed. The entire sampling process yielded 483 complete cases that were used in the analyses. That is, we followed a quantitative method to analyse the data. This consisted primarily of Covariance-based Structural Equation Modelling (CB-SEM) to assess the hypothetical structural model as well as its invariance linked to parents’ gender, income, or education.

Our self-administered survey simulated the respondents’ decision making when purchasing food at fast-food restaurants affiliated to one of the most valuable fast food brands in the world [50]. We recreated the restaurant’s full menu, including nutrition facts for each of the variety of items it serves. We then integrated the menu into our survey and asked participants to order meals for themselves and their child as they normally do when visiting the restaurant. We measured parent’s unhealthy food choice for their child as a dichotomous variable [8]. We calculated the variable employing the main nutritional values (i.e., fat as %DV, sodium and calories) [51] and coded it as 0, which represents ‘healthy’ choice whilst 1 was assigned for an ‘unhealthy’ choice [8]. That criterion was used to label choices as either unhealthy when a meal choice would give more than 35% of total calories from fat, 640 mg of sodium and 600 cal, or otherwise as healthy [51]. Child food allergy was assessed using one question: “Is your child allergic to any food?” The participants were asked that question twice and in different places in the questionnaire to double-check that they certainly were aware of their child’s food allergy status. We measured restriction and pressure to eat, as attitudinal components towards childhood obesity by employing question items from the ‘Child Feeding Questionnaire’ (CFQ) [1]. The items were evaluated using a five-point Likert scale, where 5 and 1 representing ‘strongly agree’ and ‘strongly disagree’ respectively. Sample items for pressure to eat were “I have to be especially careful to make sure my child eats enough” and “If I did not guide or regulate my child’s eating, he/she would eat much less than he/she should.” Additionally, representing items for restriction “I have to be sure that my child does not eat too many sweets (candy, ice-cream, cake or pastries)” and “I have to be sure that my child does not eat too many high-fat foods.” The factorial dimensionality of parent’s attitudes towards childhood obesity, comprising of pressure to eat and restriction, was judged to be both internally consistent and valid AVE _pressure-to-eat_ = .556; CR _pressure-to-eat_ = .789; AVE _restriction_ = .566; CR _restriction_ = .793, RMESA = .048 < 0.08, SRMR = .062 < .08, □^2^/df = 1.63 < 3, NFI = .947 > .9, and CFI = .979 > .9 [52]. Finally, we conducted Harman’s single factor test to assess the common method bias (CMB) [53] for parental attitudes towards childhood obesity. The common factor yielded 26.77% of the variance, which is far less than 50%; thus, there was no threat to CMB [53]. Amos V. 24 and SPSS V. 26 were used to run the analyses.

## Results

As stated earlier, we received 483 valid responses (57% were from the US) in the study. Seventy-four per cent were mothers. Most of the respondents had a university-level degree, an associate degree or lower (61%), earning a household income of less than USD 60,000 per year (55%). Sixty-four per cent of our sample had a tendency to pick unhealthy food options for their children. Only 17 % of the participating parents had a child allergic to some type of food allergy. Based on chi-square values, no associations were found between the socio-demographic variables and parental unhealthy food choices for their children. Employing Cohen’s *d* (for effect size calculation) alongside one-sample t-test that compared mean values for restriction and pressure to eat to the neutral value, i.e., 3 (since those variable were assessed based on 5-point Likert scale), we found that the participants exhibited medium to small positive levels of attitudes regarding both pressure to eat (mean = 3.16, SD = .84, t = 4.10, *df* = 482, *P* < .001, Cohen’s *d* = 20) and restriction (mean = 3.51, SD = .79, t = 14.18, *df* = 482, *P* < .001, Cohen’s *d* = .64). Table 1 shows the main demographics, attitudes’ descriptives and parental food choices’ frequencies broken down by the U.S. vs Canadian samples.

**Table 1:** Sample description

Variable	Label/Descriptive	Country	
		Canada	The U.S.
**Parental gender**	Male	34%	19%
	Female	66%	81%
**Child has food allergy**	Yes	16%	18%
	No	84%	82%
**Education level**	Low: < degree level	25%	59%
	High: ≥ degree level	75%	41%
**Household Income**	Low: ≤ USD 60,000/CAD 80,000	59%	52%
	High: > USD 60,000/CAD 80,000	41%	48%
**Parental food choice**	Unhealthy	62%	66%
	Healthy	38%	34%
**Restriction**	Mean	3.61	3.44
	SD	0.78	0.80
**Pressure to eat**	Mean	3.46	2.93
	SD	0.70	0.87
**Sample Size**		**208**	**275**

**Fig. 2:**
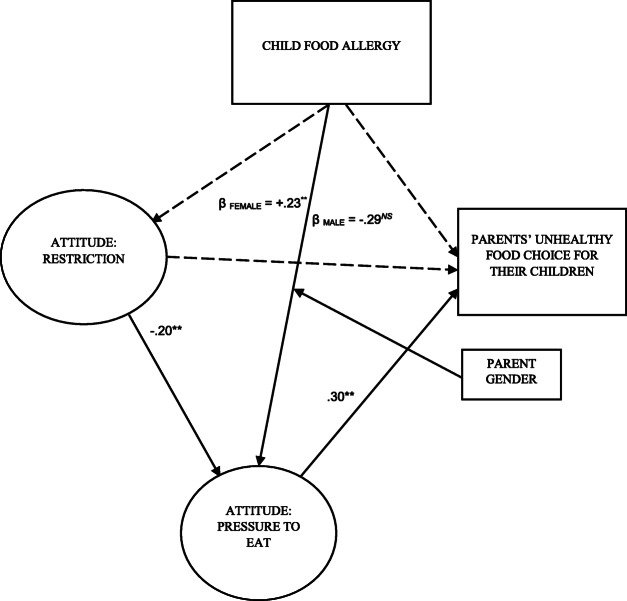
Alternate model**.** *Note. Ns* denotes non-significant; ** *p* < .01; dashed arrows represent non-significant paths

The hypothesised path (see Fig. 1) is assessed first regarding the effects of childhood food allergy on parental unhealthy food choices via pressure to eat, and restriction attitudes using IBM Amos version 25. Key fit indices are utilised to evaluate the validity of H1, H2, and H3. Although some statistics, e.g., □^2^/df = 2.86 < 3, CFI = .96 > .9, TLI = .91 > .9, SRMR = .046 < .08, RMSEA = .062 < .08 demonstrate that the path model is of an adequate fit for our sample data [8], some paths, i.e., child allergy ➔ Restriction, child allergy ➔ parental unhealthy food choices, and restriction ➔ parental unhealthy food choices were non-significant, thus, eliminated and an alternate model was produced (see Fig. 2). As shown in Table 2, the RMSEA of the alternate model dropped to 0.056 < .06, which is an indication of the improved fit. Table 3 demonstrates the testing of the indirect effects with bootstrapping and suggests that pressure to eat fully mediates the paths that link both child food allergy (B = .05; *P* < .01) and restriction (B = −.02; *P* < .01) to parental unhealthy food choices for their children. Taking all with the significance and values of estimates shown in Table 2 and Table 3 [8], we come to the conclusion that pressure to eat positively predicts parent’s unhealthy food choices for their children. Also, the pressure to eat serves as a mediator to fully transmit the positive indirect effects of childhood food allergy and the negative effects of restriction on parental unhealthy food choice for their children.

**Table 2:** Alternate model path analysis

Path			Estimate ***β***
Restriction	➔	Pressure to Eat	−.20**
Child Food Allergy	➔	Pressure to Eat	.19**
Pressure to Eat	➔	Parental Unhealthy Food Choice for their Children	.30**
*X*^*2*^*/df* = 2.51 < 3			
CFI = .958 > .9			
TLI = .933 > .9			
SRMR = .042 < .08			
RMSEA = .056 < .08			

** *P* < .01

**Table 3:** Testing indirect effects

#	Mediation path	Mediation Estimate B	Mediation type
1	Child food allergy ➔ Pressure to eat ➔ Parental unhealthy food choice	.05**	Full
2	Restriction ➔ Pressure to eat ➔ Parental unhealthy food choice	−.02**	Full

** *P* < .01

To assess the invariance of the model concerning the different levels of parent’s education and income, separate tests are also run for each of the two subgroups in parent’s education (i.e., low educated and highly educated), gender (i.e., male and female) and household income (i.e., low and high), with the outcomes being invariant from that of analysis of the sample as a whole [8]. The invariant/equivalence analysis examines the non-equivalence between an unconstrained model, which hypothesises that the groups are causing different scores of the parameters when we apply the model to the observed data, and a set of constrained models, which presume that the groups are generating invariant scores of given sets of parameters when we apply the model to the data [8]. The unconstrained model, in our case, generated a substantial chi-square value, χ^2^ (6, *N* = 483) = 19.76, *p* = .003, for parent’s gender as well as a non-substantial chi-square values regarding household income χ^2^ (6, *N* = 483) = 4.27, *p* = .641, and parent’s educational level χ^2^ (6, *N* = 483) = 8.03, *p* = .236. Thus, we come to the conclusion that parent’s gender, at least, moderates, one path in our hypothesised model. Further, in order to determine the moderated path(s), we make pairwise parameter comparisons [54]. Z score is determined based on the Bonferroni corrected level of statistical significance, which is equal to .02 [54]. Hence, we evaluate the substantiality of the pairwise parameter variations based on a Z score equal to 2.054. The findings demonstrate that only the link between child food allergy and pressure to eat is not invariant between fathers and mothers (Z = 3.279 > 2.054). Put it another way, only females, i.e., mothers, are more likely to put more feeding pressure on their food-allergic children (*β*_*mothers*_ = .23, *P* < .01). Whilst, that situation is unlikely to happen (*β*_*Fathers*_ = −.29, *P* > .05) amongst male parents (i.e., fathers). Based on these analyses (see Fig. 2), we decide that H1, H3, and H4 are partially valid. H2 is rejected.

## Discussion

This study examined a number of relationships among the theoretically proposed set of variables and found partial support for H1, H3, and H4. That suggested that pressure to eat is the main direct predictor that links positively to (and explain the changes in) parental unhealthy food choices for their children. Further, the pressure to eat serves as a full mediator, transmitting the positive indirect effects of childhood food allergy on mothers’ unhealthy food choices for their children. Furthermore, the pressure to eat was found to fully buffer the negative indirect impacts of food intake restriction on both fathers’ and mothers’ unhealthy food choices for their children. These findings are in line with previous research proposing the need for greater parental supervision regarding children’ healthy eating choices [55]. However, in times of crisis and food shortages, such as during the 2020 coronavirus (covid-19) pandemic, parental supervision may be constrained by the difficulties in accessing appropriate food, e.g. gluten-free diet [13, 56]. In addition to the empty shelves reported by Athas, CBS News [56] found that some parents experienced difficulties going out to shop due to the lockdown, and this places them at a more considerable disadvantage with regards to food purchases. This leads to some parents (particularly those on a low income with no means of transportation) diminished control over the diet of their children with allergies. This social category is generally the least prepared for times of pandemic [57]. Equally, protracted social isolation causes issues of weight gain and obesity [2, 16–18].

Interestingly, we found partial support for our H4, suggesting that mothers are more likely to be more engaged in pressure-to-eat behaviours towards their food-allergic children than fathers. This finding is in line with studies of mothers and fathers of children with a food allergy or even other health conditions (e.g., [20]). It may indicate to the propensity for many fathers and mothers to engage differently in child-related health care. In this regard, we argue that mothers seem to be keener to have their food-allergic children on a full stomach than fathers do. Such keenness appears to be embodied with making children with food allergy eat more of their ‘free from’ food (i.e., free from relevant allergens). Thus, these children would not feel the desire to eat other non-especially-prepared food, that might contain allergens, in the absence of the parents.

Our results indicate that more efforts are required to educate parents about their children’s healthy food decisions. The study also suggests that during a crisis period such as the current on-going covid-19 pandemic, families with children with allergies need more information and support in order to maintain certain normality in the diet of their children to curb anxiety and poor health. Such support will give parents the confidence to allergy reactions more effectively [14] and improve their own mental wellbeing [15]. While governments and medical practitioners are determined to combat the increasing prevalence of childhood obesity, the results of this research indicate that they are fighting an uphill battle. Not only is compulsive and unhealthy eating prevalent in society; it has become an epidemic. Thus, health and nutritional information of the meals should be communicated to parents more effectively, i.e., in a simple, understandable and accessible way and using a wide variety of communication channels to reach all social groups. Future research can tackle the need for preventive actions rather than a cure. In particular, future researchers can investigate how marketing activities could aid parents to prevent obesity in children rather than seeking a cure after the epidemic has occurred.

A potential role for the public policy regarding parental healthier food decisions for their children could be implemented at the individual level (i.e., the parents as customers) through taxing unhealthy meals at fast-food restaurants. Taking such a measure may entail taxing foods high in calories, saturated fat, sodium, and sugar, or, in other words, taxing the causes of dietary obesity as that would reduce the affordability of unhealthy foods [58]. However, that should be done with caution since such an approach is likely to be regressive, i.e., poor people would pay a higher share of their income taxes than rich people would [59]. The implementation of a food tax is not a new policy on public health for unhealthy food products. Finland, France and Hungary alone implemented a new tax on soft drinks in 2011 and 2012. Similarly, there has been a ‘sweet’ tax imposed on soft drinks in the city of Berkeley (California) [60] and the city of Philadelphia (Pennsylvania) in the USA [61]. In parallel, Denmark introduced a new tax on foods containing more than 2.5% of saturated fat content [59]. In this regard, many researchers have suggested that introducing such taxes has resulted in significant improvements in people’s dietary intake [62].

While we conducted this study in two Northern American countries of the United States and Canada, the results should be of sufficient external validity in other states within both countries. The data collected from two small regions within the US and Canada are generalisable to regions beyond those sampled in the present study, considering the widespread nature of childhood obesity [63]. However, further replications should be performed to validate our theoretical model either in developed countries or other contexts. Such studies would educate health care professionals, governments, and parents of any variances between cultures, ethnic groups or social classes regarding making unhealthy food choices for children [64].

The use of self-reporting data, which, can generate bias in the responses obtained. It is because this sort of data implicitly suggests that the participants have the same understanding or interpretation of the questioning [65]. Some researchers criticise the use of the cross-sectional design in concluding causal relationships. Nonetheless, findings generated from a cross-sectional study can still be regarded as interpretable and valid as long as they are conducted on a sound theoretical basis [66]. Since there is a limited theoretical basis, the conclusions about possible causality of the relationships studied in this research, however, carrying out future research to assess the validity of the existing model using a longitudinal design would be significantly endorsed.

Furthermore, the inclusion of objective assessments of variables that could relate to childhood obesity, e.g., Body Mass Index (BMI) would be highly endorsed while either replicating our study or generating a new model. It is worth noting that such variables could further moderate the relationships amongst the variables under investigation. For example, parental attitudes towards obesity may predict the child’s weight status. Additionally, we built our methodology on a set of specific criteria to assess the healthiness of meal choice. The conditions were based on calories, sodium, and fat as %DV [51]. As a result, a meal judged as healthy by criteria other than those utilised in this study can be decided unhealthy against the criteria we adopted in this study. Thus, we suggest that future research replicates our study alongside adopting more inclusive criteria (or different ones) to assess meal healthiness.

The pressure to eat has been linked to increased maternal concern about a child being ‘underweight’ [67], as well as children’s food fussiness [68], and lower Body Mass Index (BMI) percentile scores in children [41]. All of which could be considered as variables of interest in future studies to develop more sophisticated models that would advance our knowledge of parental food choice. While our study models a path from childhood allergy to unhealthy food choices via attitudes, we conceptualised parent’s ‘gender’ as a moderating variable rather than an exclusion criterion, which creates avenues for future investigations.

Further research is advised to assess what we would call ‘parent-child meal consultation’ could affect parental more democratic choices of healthy food for their children, especially those with food allergy. Finally, the approach of using a fast-food menu, which would offer limited healthy options, can bias the responses and data collected. It would add to the existing knowledge base if a similar study could capture the differences between parents who frequent fast food places against those who only order there on occasion, to gauge the degree of impact on food choices. Future research is encouraged to help understand choices based on interest, taste, preference, healthfulness, allergen avoidance, and food group avoidance (e.g., vegetarians). There has been evidence of the role of child gender (though not assessed within the scope of our study) in eating behaviour and parent-child dynamics [69]. Thus, to develop a full picture of parental food choices, additional studies will be needed that child gender would be considered as a covariate.

## Conclusion

We investigated a theoretically proposed conceptual model predicting parental unhealthy food choices. We found that pressure to eat, positively predict parental unhealthy food choices for their children. Additionally, it transmits the childhood food allergy indirect effects on mothers’ unhealthy food choices for their children. It makes such poor food selections more likely in case of the presence of childhood allergy. Parental engagement in pressure to eat behaviour was found to play another mediating role. It blocks the chances of engaging in healthy parental food choices because of relating to fathers’ and mothers’ tendency to set restrictions on their children’s food intake. The paper contributes to the literature on food allergies among children and the marginalisation of families with allergies. Our explorative model is a first of its kind and highlights the complex relationships between the variables under consideration. Though the data collection precedes the covid-19 pandemic, our findings are relevant to the pandemic era because if in ordinary time families with allergies face significant challenges for making adequate food choices, then crisis periods such as the covid-19 pandemic could exacerbate the difficulties for those families. The paper, therefore, bears unique significance in consequence of the critical period in which it took place, i.e. the global coronavirus (Covid-19) pandemic as it draws the attention of policymakers to the vulnerability of families with allergies and how should also be prioritised in crisis times.

## Data Availability

The data used in this study will not be available due to confidentiality issues.

## Abbreviations

StatCan: Canadian Community Health Survey
Covid-19: Coronavirus pandemic
